# Metabolic Cytokines at Fasting and During Macronutrient Challenges: Influence of Obesity, Female Androgen Excess and Sex

**DOI:** 10.3390/nu11112566

**Published:** 2019-10-24

**Authors:** M. Ángeles Martínez-García, Samuel Moncayo, María Insenser, Francisco Álvarez-Blasco, Manuel Luque-Ramírez, Héctor F. Escobar-Morreale

**Affiliations:** Diabetes, Obesity and Human Reproduction Research Group, Department of Endocrinology & Nutrition, Hospital Universitario Ramón y Cajal & Universidad de Alcalá & Instituto Ramón y Cajal de Investigación Sanitaria IRYCIS & Centro de Investigación Biomédica en Red Diabetes y Enfermedades Metabólicas Asociadas CIBERDEM, Madrid 28034, Spain; manitamg@yahoo.es (M.Á.M.-G.); samuel.moncayo@gmail.com (S.M.); mariarosa.insenser@salud.madrid.org (M.I.); fablas74@gmail.com (F.Á.-B.); manuluque@gmail.com (M.L.-R.)

**Keywords:** adipokines, PCOS, sex hormones, adiposity, glucose, lipids, proteins, oral loads

## Abstract

Scope: Cytokines have pleiotropic functions within the organism and their levels may be influenced by obesity, visceral adiposity and sex hormones. Diet composition may also affect their systemic concentrations during fasting and in the postprandial period. Hence, we studied the influence of sex steroids and obesity on the circulating levels of a panel of metabolic cytokines in the fasting state and after single macronutrient challenges. Methods: On alternate days we submitted 17 women with polycystic ovary syndrome (PCOS) (9 non-obese, 8 obese), 17 non-hyperandrogenic control women (9 non-obese, 8 obese) and 19 control men (10 non-obese, 9 obese) to isocaloric oral glucose, lipid and protein loads. Serum levels of omentin-1, vaspin, lipocalin-2, adipsin, PAI-1, chemerin, FGF-21 and FGF-23 were determined by Luminex multiplex technology. Results: During fasting, obese patients presented higher levels of PAI-1, chemerin and adipsin but decreased FGF-23 and omentin-1 compared with non-obese subjects. Vaspin showed sexual dimorphism with lower levels in men than women with PCOS and female controls. Following macronutrient ingestion, most metabolic cytokines presented a similar physiological response consisting of a decrease in circulating concentrations, which was inversely associated with the fasting levels of these molecules. Protein intake caused the major postprandial decrease whereas glucose did not significantly reduce PAI-1, FGF-23 and vaspin, and even increased FGF-21. Regardless of the macronutrient administered, vaspin levels showed a larger reduction in non-obese individuals while the decrease in PAI-1 was particularly noticeable in the obese subgroup. The postprandial reductions of omentin-1 and FGF-23 after glucose and protein loads were influenced by obesity. No major differences were found between patients with PCOS and male and female controls. Conclusions: Obesity, but not PCOS or sex, markedly influences metabolic cytokine levels at fasting and after macronutrient ingestion. The observed postprandial decrease in their circulating concentrations might represent a physiological compensatory mechanism against food-induced inflammation and oxidative stress. This mechanism is altered by obesity and is differently modulated by macronutrients, suggesting a larger contribution of glucose to stressful postprandial responses.

## 1. Introduction

Cytokines have pleiotropic functions within the organism including the modulation of immune inflammatory responses and the regulation of food intake and energy balance by acting in the central nervous system and in peripheral tissues [1]. These metabolic hormones are produced in diverse organs and tissues such as adipose, liver and bone that secrete adipokines and related molecules with critical roles in inflammation, insulin sensitivity and carbohydrate and lipid metabolism. Reciprocally, systemic levels of these cytokines may also be affected by dietary factors and meal ingestion [2,3].

Circulating adipokine concentrations—both in the fasting state and after an oral glucose challenge—are not only influenced by weight excess but also by abdominal obesity, sex and sex hormones [4,5]. In this regard, patients with polycystic ovary syndrome (PCOS), which is the most prevalent endocrine and metabolic disorder in premenopausal women [6], may present different cytokine levels compared with non-hyperandrogenic women [7]. Although androgen excess is possibly the pivotal pathogenic mechanism in PCOS, obesity, abdominal adiposity, adipose tissue dysfunction and insulin resistance are frequently shown in these women [8]. As other individuals with adipose tissue and metabolic dysfunction, PCOS patients manifest postprandial dysmetabolism—a sustained elevation of carbohydrates and lipids in the postprandial period [9]—that has also been associated with impaired cytokine secretion [10].

The extent to which changes in energy balance impact several circulating cytokine levels has been previously examined but most of the works studied the long-term effects of diets with different macronutrient composition during fasting or after administration of high-calorie mixed meals [3,11,12,13,14,15]. In contrast, the contribution of sexual steroids and the acute physiological consequences of isolated macronutrient ingestion, particularly proteins, have not been investigated as much [16,17]. Dandona and colleagues [18,19,20,21,22] comprehensively addressed the role of glucose and lipids in promoting postprandial inflammation and oxidative stress. In addition, several studies by Gonzalez et al. evaluated such responses in patients with PCOS after glucose and cream ingestion [23,24,25,26].

We hypothesized that sex hormone imbalances and body weight may influence the secretion of metabolic cytokines regulating energy and nutrient metabolism, and that these differences may also depend on the macronutrient being ingested. Therefore, we studied in a series of young healthy subjects, composed of non-obese and obese women with PCOS and appropriate male and female controls, the circulating levels of a panel of metabolic markers—including six adipokines and two members of the fibroblast growth factor (FGF) superfamily—during fasting and after glucose, lipid and protein oral challenges.

## 2. Materials and Methods 

### 2.1. Subjects

This report is part of a broader study addressing postprandial metabolism as a whole in young adults, parts of which have been already reported [27,28]. The 53 individuals included in the whole project consisted of consecutive women with PCOS who were recruited immediately after being diagnosed with this condition at the Department of Endocrinology and Nutrition of Hospital Universitario Ramón y Cajal, and of healthy non-obese and obese volunteers recruited from the hospital’s staff and by noticeboard advertising. Volunteers, who received a small economic compensation for local travel and other minor expenses, were selected so that they were similar in terms of age and body mass index with the women with PCOS. The subjects were also subgrouped into non-obese (BMI < 30 kg/m^2^, *n* = 28) and obese (BMI ≥ 30 kg/m^2^, *n* = 25). PCOS was defined by the presence of clinical and/or biochemical hyperandrogenism, menstrual dysfunction, and exclusion of other disorders [29]. Hypogonadal men—defined by a circulating total testosterone concentration < 8 nmol/l—were also excluded. Before enrollment, the participants had not been diagnosed with obesity-associated comorbidities such as disorders of glucose tolerance, hypertension or cardiovascular events. None of the participants were smokers nor had a history of infertility, ovariectomy, hysterectomy or had received treatment with oral contraceptives, antiandrogens, or insulin sensitizers for at least 6 months before sampling. The study was approved by the local ethics committee and all participants gave written informed consent (PI1100357).

### 2.2. Study Design

All individuals underwent a comprehensive clinical, anthropometric and physical evaluation. The protocol for oral macronutrient challenges has been described elsewhere [28]. Participants were instructed to follow a carbohydrate-unrestricted diet (at least 300 g of carbohydrates per day during 3 days) for 72 hours before sampling in order to avoid false positive results in the 75 g oral glucose tolerance test (OGTT), and were submitted, on alternate days, to the oral macronutrient loads in the following order: glucose, lipids and proteins. Challenges could not be randomized due to the need for performing in the first visit the hormonal analyses for diagnosis and the OGTT after the three-day diet. The quantities and volumes in each load were adjusted for a total calorie intake of 300 kcal. Detailed information of the protocol and macronutrient load composition is available in Supplementary Data Text.

### 2.3. Laboratory Measurements

Blood samples were obtained after a 12 h overnight fasting (during the follicular phase of the menstrual cycle in women) and during the postprandial phase (60 and 120 min after ingestion of the glucose and protein loads, or 120 and 240 min after the ingestion of the lipid load considering the slower intestinal absorption rate of lipids). Aliquots were immediately assayed or frozen at −80 °C. These samples were used for the measurement of total testosterone (T), total estradiol (E_2_), sex hormone-binding globulin (SHBG), glucose, insulin, high-sensitivity C-reactive protein (hsCRP) and lipid profile. Technical characteristics of these assays have been described in detail elsewhere [30,31]. Fasting glucose and insulin levels were used for the homeostasis model assessment of insulin resistance (HOMA-IR) [32] and the composite insulin sensitivity index (ISI) was estimated from the glucose and insulin concentrations measured during the OGTT [33].

### 2.4. Multi-Analyte Profiling of Metabolic Cytokines

We selected a panel of metabolic markers with known functions in nutrient metabolism, energy homeostasis and inflammation including six adipokines (omentin-1, vaspin, chemerin, lipocalin-2, adipsin and PAI-1) [34,35,36,37,38,39], and two members of the FGF superfamily (FGF-21 and FGF-23) [40,41]. Multi-analyte profiling was performed in serum samples on the Luminex Magpix system (Luminex Technologies, Austin, USA) and data were analyzed with the Milliplex Analyst software version 5.1 (EDM Millipore Corporation, Massachusetts, USA). The multiplex kits used and their assay characteristics are detailed in Supplementary Data Text.

### 2.5. Sample Size Analysis

We used the online sample size and power calculator provided by the Institut Municipal d’Investigació Mèdica from Barcelona, Spain, version 7.12 (https://www.imim.cat/ofertadeserveis/software-public/granmo/, last accessed June 7, 2019). Calculations were based on previously reported differences in PAI-1 concentrations observed between non-obese and obese subjects [42] and between women with and without PCOS [43]. Setting alpha at 0.05 and beta at 0.10 for a two-sided test, the inclusion of nine subjects per group would permit the detection of a difference in the means between non-obese and obese subjects of 12.1 ng/mL, assuming a standard deviation of 7.3 ng/mL [42]. Ten patients per group would allow for detecting a difference in the means of control women and patients with PCOS of 52 ng/mL, assuming a standard deviation of 33 ng/mL [43].

### 2.6. Statistical Analysis

Results are expressed as mean ± SD (tables) or mean ± SEM (figures). Normality of continuous variables was tested by the Kolmogorov-Smirnov method and logarithmic transformation was applied as needed. Two-way univariate general linear models (GLM) served to analyze within a single analysis the influence of group (i.e., control women, women with PCOS and men), obesity and the interaction of both factors on clinical and fasting biochemical variables, while adjusting the level of significance to compensate for the multiple comparisons involved. Univariate repeated-measures GLMs were used to compare postprandial concentrations of metabolic cytokines from fasting levels in each macronutrient challenge. Areas under the curve (AUC) were calculated using the trapezoidal rule (normalized by the duration of oral loads) to compare the responses among the different macronutrient loads. We introduced the AUCs as within-subjects factor in univariate repeated-measures GLM that considered also group and obesity as between-subjects factors. Mauchly’s test was used to estimate sphericity and the Greenhouse-Geisser correction factor was applied when needed. The least significant difference (LSD) *post hoc* test was used for multiple comparisons. Relationships between continuous variables were analyzed by Pearson’s correlation analysis. Analyses were performed with SPSS Statistics 15.0 (SPSS Inc., Chicago, IL, USA) and *p* values < 0.05 were considered statistically significant.

## 3. Results

### 3.1. Effect of Group and Obesity on Clinical and Biochemical Variables in the Fasting State

The clinical, hormonal and metabolic characteristics of participants are summarized in Table 1. Briefly, patients with PCOS presented both clinical and biochemical hyperandrogenism but did not show higher waist circumference (WC), waist to hip ratio (WHR) or differences in metabolic parameters when compared with control women. Besides their higher T levels, men also presented increased WC and WHR but lower levels of high-density lipoprotein cholesterol (HDL) and SHBG compared with both groups of women. Obesity was characterized by increased levels of free T, total and free E_2_, hsCRP, fasting insulin and glucose, greater WC, WHR and HOMA-IR, and by decreased SHBG and ISI value.

Serum concentrations of metabolic cytokines in the fasting state are shown in Table 2. Regardless of the group of subjects, obese patients presented higher circulating levels of PAI-1 and chemerin but decreased FGF-23 and omentin-1 concentrations compared with non-obese individuals. A similar tendency towards increased adipsin concentrations in obese patients was close to reach statistical significance (Table 2).

On the other hand, men showed lower vaspin levels compared to patients with PCOS and female controls (Table 2). We also observed an interaction between group of subjects and obesity on lipocalin-2 concentrations (Table 2): in non-obese subjects, PCOS patients showed the highest values and control women the lowest, while in obese patients, obese control women had increased lipocalin-2 levels compared with their non-obese counterparts, an effect that was not present either in patients with PCOS or in men (Table 2).

### 3.2. Effect of Oral Macronutrient Challenges on Metabolic Cytokine Concentrations

**Figure 1:**
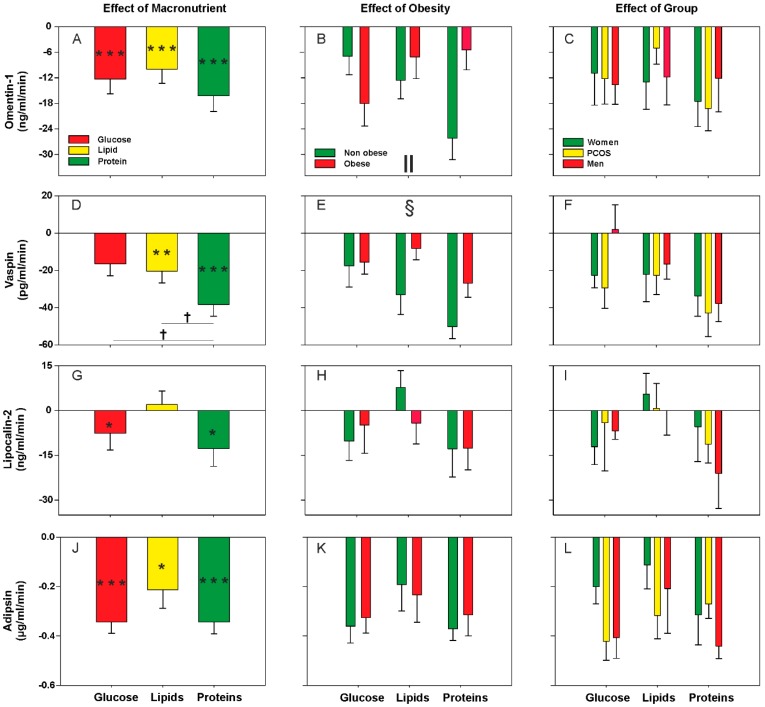
Areas under the curve (AUC) of circulating Omentin-1, Vaspin, Lipocalin-2 and Adipsin during oral macronutrient challenges considering all subjects as a whole, and according to obesity and group of subjects. Data are means ± SEM. *P* values represent the statistical significances of the differences within (introducing the three values along the curve as within-subjects factor) and between (introducing the AUC of each load as within-subjects factor and group and obesity as between-subjects factors) macronutrient loads analyzed by repeated-measures general linear models. *p < 0.05, **p < 0.01 and ***p < 0.001 for differences from fasting levels within macronutrient load. †*p* < 0.05 for the differences among the glucose, lipid, and protein loads, regardless of obesity and group of subjects. §*p* < 0.05 for the global effect of obesity, irrespective of macronutrient and group of subjects. ‖*p* < 0.05 for the interaction between macronutrient and obesity, regardless of group of subjects.

**Figure 2:**
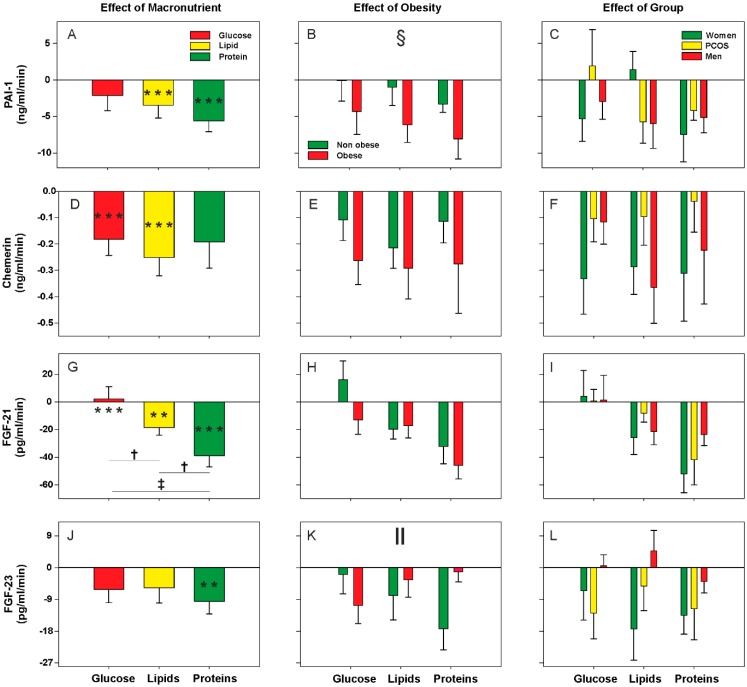
Areas under the curve (AUC) of circulating PAI-1, Chemerin, FGF-21 and FGF-23 during oral macronutrient challenges considering all subjects as a whole, and according to obesity and group of subjects. Data are means ± SEM. *P* values represent the statistical significances of the differences within (introducing the three values along the curve as within-subjects factor) and between (introducing the AUC of each load as within-subjects factor and group and obesity as between-subjects factors) macronutrient loads analyzed by repeated-measures general linear models. ^**^p < 0.01 and ^***^p < 0.001 for differences from fasting levels within macronutrient load. ^†^*p* < 0.05 and ^‡^*p* < 0.01 for the differences among the glucose, lipid, and protein loads, regardless of obesity and group of subjects. ^§^*p* < 0.05 for the global effect of obesity, irrespective of macronutrient and group of subjects. ^‖^*p* < 0.05 for the interaction between macronutrient and obesity, regardless of group of subjects.

Considering all subjects as a whole, we observed a decrease or small response of metabolic cytokine levels during the postprandial state, without large differences among the distinct macronutrient loads (Figure 1A,D,G,J and Figure 2A,D,G,J). Exceptions to this rule were protein intake causing the largest effect on decreasing circulating cytokine concentrations whereas, on the contrary, glucose ingestion failed to reduce vaspin, PAI-1 and FGF-23 levels and even caused an increase in FGF-21 concentrations (Figure 1D and Figure 2A,G,J). Accordingly, after the oral protein challenge, the negative AUCs of vaspin and FGF-21 were larger than those observed after the lipid and glucose oral loads (Figure 1D and Figure 2G). The oral lipid challenge caused a decrease in the circulating levels of all metabolic cytokines studied here with the exception of lipocalin-2 (Figure 1G) and FGF-23 (Figure 2J). 

Considering all individuals as a whole, we observed a global effect of obesity on postprandial vaspin and PAI-1 levels regardless of the macronutrient being administered. Vaspin concentrations showed a greater reduction in non-obese subjects compared with obese patients (Figure 1E). Conversely, the decrease in PAI-1 levels was particularly noticeable in the obese subgroup (Figure 2B). Omentin-1 and FGF-23 levels showed significant interactions between obesity and macronutrients because obesity influenced postprandial levels differently during the oral glucose and protein challenges. Compared with their non-obese counterparts, obese subjects presented a much smaller response of both cytokines after protein ingestion—the postprandial reduction described earlier was mainly attributable to the non-obese subgroup—whereas opposite changes were observed after glucose ingestion (Figure 1B and Figure 2K). Finally, we found no statistically significant differences between patients with PCOS and female and male controls in the responses of any metabolic cytokine (Figure 1C,F,I,L and Figure 2C,F,I,L), nor any interaction of the group of subjects with obesity or macronutrient intake (data not shown).

In addition, we found that fasting levels of the studied molecules showed inverse correlations with their corresponding AUCs in response to macronutrient intake almost universally, although for some cytokines these correlations were influenced by obesity (Table 3).

An overview of the effects of obesity and group of subjects on metabolic cytokine levels in the fasting and postprandial states is presented in Table 4.

## 4. Discussion

Our present results indicated that obesity exerted a major influence on circulating levels of several cytokines in the fasting state, in contrast to the much smaller impact of sex and female androgen excess. Following macronutrient ingestion, we observed a similar physiologic response in most metabolic cytokines studied here consisting of a decrease in their circulating concentrations that was inversely associated to their fasting levels. Overall, the lowering effect was greater after protein ingestion and less pronounced after the glucose load, which caused no changes or even small increases in some cytokines. As occurred for the fasting state, these postprandial responses were not influenced by sex or female hyperandrogenism but were modulated by obesity, suggesting that the postprandial changes occurring after macronutrient intake in healthy individuals are deregulated in obese subjects.

Weight loss, following either dietary restriction or bariatric surgery of individuals ameliorates the proinflammatory and prooxidant state associated with obesity [44,45,46,47,48]. Earlier studies addressed the association of metabolic cytokines with obesity during fasting [34,36,37,39,49]. Our results, obtained from a series of young and otherwise healthy subjects, showed that obesity influenced circulating levels of many of the cytokines evaluated here at fasting, resulting in increased circulating concentrations of PAI-1, chemerin and adipsin and reduced levels of omentin-1 and FGF-23, when compared with non-obese individuals. Lipocalin-2 was higher in obesity but only in control women; whereas in the non-obese subgroup patients with PCOS presented the largest concentrations. On the contrary, vaspin showed sexual dimorphism consisting of decreased levels in men compared with women, as has been previously described in children and adolescents [50].

The association of FGF-23 concentrations with obesity and insulin resistance is unclear [51,52,53,54], possibly because some studies reporting positive associations with BMI [51,55] measured total FGF-23 (full-length and C-terminal fragments) instead of measuring intact FGF-23 as we have done (Supplementary Data Text). In contrast, intact FGF-23 levels were increased in non-obese subjects in our study.

Of the metabolic cytokines studied here, we only found that lipocalin-2 concentrations were increased in non-obese women with PCOS compared with female controls. The study of these circulating molecules on PCOS yielded largely discrepant results, not only with our present data, but also among earlier reports [56,57,58,59,60,61,62,63,64,65,66,67,68,69,70,71]. Such discrepancies might reflect the heterogeneity and complex nature of PCOS, but also differences among studies in the criteria used to diagnose the syndrome and in the characteristics of the population in terms of the association with obesity and other confounding factors which influence heavily metabolic variables. Our study design and statistical analysis allowed the independent evaluation of the effects of obesity and of group but also their interaction, and the fact that our PCOS patients were not more insulin resistant than the control groups also eliminated this variable as a putative confounding factor. For example, a previous study reported that PAI-1 levels were increased in women with PCOS compared with control women, but detailed analysis found that insulin, HOMA index and BMI, and not androgen excess, were the variables actually associated with PAI-1 antigen levels [72].

Many of the studies addressing the role of diet components on physiological and metabolic changes during the postprandial phase have used high-calorie mixed meals rich in carbohydrates and fats [73,74,75,76] hampering the precise contribution of carbohydrates, lipids and proteins to these changes. Instead, with the aim of providing data representative of a physiological postprandial response, we chose isolated glucose, lipid and protein challenges conducted after overnight fasting, in the amounts needed to provide a modest calorie intake of 300 kcal, which is close to that of a regular breakfast [77,78].

Overall, circulating concentrations of metabolic cytokines decreased during the postprandial phase, and such a decrease was larger after protein ingestion than after lipid and glucose oral administration, even though these differences only reached significance for vaspin and FGF-21. Of note, during the oral glucose challenge the levels of PAI-1, vaspin and FGF-23 did not decrease, and even increased in the case of FGF-21. The fact that the postprandial decrease was inversely proportional to the levels observed at fasting in most of these metabolic mediators suggests a physiological compensatory response aiming to avoid a further increment in their levels. Lipocalin-2 did not show a postprandial reduction following lipid ingestion, a finding that could be related to the known effect of fats increasing gut permeability [79]. Changes in intestinal permeability and dysbiosis might contribute to systemic inflammation and insulin resistance [80] and, because lipocalin-2 has an impact on gut microbiota and mediates intestinal and systemic inflammation, the stability of its levels after lipid ingestion might contribute to postprandial intestinal homeostasis [37].

Consistent with our present data, most studies addressing circulating PAI-1 levels after high fat meals showed a postprandial decrease in healthy non-obese men [74], in obese subjects [75] and in patients with type 2 diabetes [76]. Similarly, circulating FGF-21 concentrations decreased after oral lipid administration but not during an oral glucose tolerance test in two large cohorts of healthy individuals [81]. In fact, serum FGF-21 levels actually increased after glucose ingestion in our study. The predominant role of FGF-21 in the adaptive responses to nutritional and physiological stressors [40] consists of ameliorating postprandial endoplasmic reticulum stress [82] and glucose is the major responsible of inflammatory and oxidative stress postprandial changes [27,28], further suggesting a compensatory homeostatic mechanism.

Previous studies suggested that the postprandial decrease in serum FGF-21 levels observed after oral fat administration might be related to postprandial triglyceride-rich lipoproteins and to liver fat [83]. Likewise, chemerin and adipsin decreased after an oral lipid challenge in a putative anti-inflammatory adipokine response aiming to counteract the proinflammatory effects of postprandial hyperlipidemia [84]. However, in our study we observed an even greater decrease after protein intake suggesting that other regulatory mechanisms may be implicated. Indeed, we have recently reported similar postprandial responses in circulating inflammatory markers [27] and energy homeostasis mediators during macronutrient challenges (data submitted for publication), and other authors have observed that energy intake itself had a suppressive effect on adipokine concentrations [85].

We may hypothesize that the postprandial decrease in these molecules is consequence of physiological compensatory mechanisms with differences that may rely on the specific capacity/ability of each macronutrient to produce distinct metabolic, hormonal, inflammatory or oxidative stress responses in diverse cells and tissues. Therefore, the smaller decrease observed for some cytokines after glucose load might be related to its triggering effect on inflammation and oxidative stress [3,10,11,18,19,21,27,28,40]. In contrast, the protein load caused the smallest induction of leukocyte gene expression of inflammatory mediators [27] and did not increase plasma TBARS levels [20,28], and thus it is conceivable that, in absence of other stimulus, the greatest decrease of circulating concentrations is probably due to other physiological regulatory processes/mechanisms that require nutrient transit through the gastrointestinal tract, like those exerted by bile acids and gut hormones [80,86,87]. Lipid ingestion had intermediate effects between glucose and proteins that might be explained by the large percentage of mono- and polyunsaturated fatty acids in our lipid emulsion, since saturated and unsaturated fats have been reported to induce or protect from inflammation, respectively [88], and many of the studies reporting a proinflammatory and prooxidant effect of lipids actually used saturated fats [19,20].

In addition, we observed that for some cytokines this postprandial physiologic process is impaired in obesity regardless of sex or female androgen excess. Independently of the macronutrient ingested, obese subjects presented a blunted postprandial response of vaspin concentrations but a large decrease in PAI-1 levels. Our results also revealed that obesity modulated the response of omentin-1 and FGF-23 to the different macronutrients. Non-obese subjects showed a small response to oral glucose intake and a larger decrease after protein ingestion, whereas the opposite was observed in obese patients. Accordingly, the correlations between fasting levels and AUCs of macronutrient loads were different in the non-obese and obese subgroups. These results further support the hypothesis of deregulation of postprandial physiologic compensatory mechanisms with obesity, as we have recently proposed for other inflammatory and energy homeostasis mediators like IL-18, ghrelin and leptin [27] (data submitted for publication).

Very little is known about the influence of sex and PCOS on the changes of these metabolic mediators during the immediate postprandial state. A very recent study evaluating a panel of adipokines such as PAI-1 and adipsin after an oral glucose challenge reported postprandial decreases for both molecules that were similar in women with PCOS and female controls [60]. In agreement, we have not found differences in the responses to any macronutrient challenge among patients with PCOS, female controls and men.

The latter might be related to certain limitations of our study. The sample size of the subgroups was relatively small and we only obtained samples at two postprandial time points given the complicated logistics involved; hence, our study may have been underpowered to detect small differences among groups of subjects and might have missed changes requiring a longer period of time after ingestion. We did not include an isovolumetric water control to test for confounding factors (e.g., hemodilution or diurnal fluctuations in serum concentrations), and the order of macronutrient challenges was not randomized, yet the possibility of carryover effects was limited by the modest calorie content of the oral loads and minimized by conducting the challenges in alternate days. In our humble opinion, these limitations were compensated by the rather homogeneous healthy population studied here in terms of age and percentage of obesity, the quality of the procedures used in the challenges, the administration of exactly the same number of calories in the three oral loads, and the recommendation made to all subjects of following the same diet for a few days before the start of the study.

## 5. Conclusions

Our present results, derived from young healthy adults, suggest that food-induced inflammation and oxidative stress are counteracted by postprandial physiologic mechanisms that decrease the concentrations of metabolic cytokines, and that obesity impairs such a response. These compensatory responses appear to be also impaired after glucose ingestion, at least when compared with oral protein intake. On the contrary, neither sex nor female androgen excess exerted major effects on fasting and postprandial concentrations of metabolic cytokines. Precise knowledge of the postprandial effects of the distinct macronutrients may permit tailoring the dietary management of subjects who already present or are susceptible to suffer from low-grade chronic inflammation and increased oxidative stress, such as obese individuals and women with PCOS.

## Figures and Tables

**Table 1:** Clinical, biochemical and hormonal characteristics of participants.

	Control Women		Women with PCOS (Polycystic Ovary Syndrome)		Control Men		Group	Obesity	Interaction
	Non-obese (*n* = 9)	Obese (*n* = 8)	Non-obese (*n* = 9)	Obese (*n* = 8)	Non-obese (*n* = 10)	Obese (*n* = 9)	*P*	*P*	*P*
Age (years)	26 ± 5	27 ± 6	24 ± 8	30 ± 4	24 ± 5	25 ± 4	0.342	0.101	0.319
Body mass index (kg/m^2^)	23 ± 2	36 ± 4	24 ± 2	37 ± 5	23 ± 2	34 ± 3	0.226	<0.001	0.782
Waist circumference (cm)	76 ± 9	100 ± 17	72 ± 7	105 ± 11	81 ± 5	110 ± 13	0.049 ^B,C^	<0.001	0.377
Waist to hip ratio	0.75 ± 0.08	0.83 ± 0.12	0.73 ± 0.05	0.85 ± 0.06	0.83 ± 0.04	0.90 ± 0.05	0.002 ^B, C^	<0.001	0.436
Hirsutism score	1.4 ± 1.3	1.8 ± 1.2	9.7 ± 4.5	9.3 ± 4.5	-	-	<0.001 ^A^	0.738	0.876
Total testosterone (ng/dL)	46 ± 9	58 ± 14	72 ± 20	69 ± 29	534 ± 96	499 ± 104	<0.001 ^A,B,C^	0.776	0.196
Free testosterone (ng/dL)	0.6 ± 0.2	0.9 ± 0.0	1.0 ± 0.4	1.3 ± 0.7	13.0 ± 3.0	13.4 ± 2.7	<0.001 ^A,B,C^	0.024	0.265
Total estradiol (pg/mL)	41 ± 17	75 ± 55	50 ± 55	41 ± 13	19 ± 4	26 ± 7	<0.001 ^B,C^	0.024	0.422
Free estradiol (pg/mL)	0.74 ± 0.30	1.44 ± 0.82	0.98 ± 1.17	0.93 ± 0.38	0.49 ± 0.14	0.71 ± 0.19	0.010 ^B^	0.003	0.520
Ratio FT/FE_2_	8.6 ± 0.9	7.6 ± 1.6	17.4 ± 4.0	14.0 ± 1.9	263.4 ± 22.6	186.4 ± 12.7	<0.001 ^A,B,C^	0.119	0.750
SHBG (µg/dL)	504 ± 225	387 ± 126	450 ± 189	288 ± 117	243 ± 90	180 ± 54	<0.001 ^B,C^	0.008	0.568
Androstendione (ng/mL)	2.6 ± 0.8	2.7 ± 0.8	4.2 ± 1.2	3.8 ± 1.8	2.0 ± 0.5	2.7 ± 1.1	<0.001 ^A,C^	0.712	0.371
DHEAS (ng/mL)	1990 ± 442	1953 ± 553	2800 ± 1437	2100 ± 774	2211 ± 663	3021 ± 1068	0.094	0.852	0.081
hsCRP (mg/L)	2.8 ± 2.3	4.0 ± 2.9	2.1 ± 2.2	6.8 ± 7.7	3.3 ± 2.6	3.2 ± 1.3	0.998	0.009	0.207
Fasting insulin (µIU/mL)	7.9 ± 2.9	11.1 ± 3.0	7.5 ± 4.2	13.5 ± 3.6	5.8 ± 1.6	10.8 ± 3.9	0.123	<0.001	0.440
Fasting glucose (mg/dL)	85 ± 7	96 ± 7	81 ± 9	87 ± 9	88 ± 9	94 ± 7	0.011 ^A,C^	0.001	0.408
HOMA-IR	1.6 ± 0.6	2.6 ± 0.7	1.5 ± 0.9	2.9 ± 0.7	1.3 ± 0.4	2.5 ± 1.0	0.424	<0.001	0.742
Insulin sensitivity index	6.6 ± 2.9	3.3 ± 1.2	8.1 ± 4.7	3.5 ± 1.4	7.3 ± 2.8	3.8 ± 1.6	0.640	<0.001	0.909
Triglycerides (mg/dL)	75 ± 32	81 ± 30	80 ± 22	101 ± 42	79 ± 26	102 ± 35	0.399	0.054	0.711
Total cholesterol (mg/dL)	170 ± 35	181 ± 35	166± 39	170 ± 35	158 ± 23	185 ± 35	0.844	0.133	0.485
HDL-cholesterol (mg/dL)	54 ± 12	51 ± 8	54 ± 8	46 ± 8	46 ± 8	39 ± 4	0.001 ^B, C^	0.002	0.512
LDL-cholesterol (mg/dL)	104 ± 31	112 ± 23	97 ± 31	104 ± 31	93 ± 15	127 ± 31	0.507	0.034	0.237

*Abbreviations*: FE_2_, free estradiol; FT, free testosterone; HDL, High density lipoprotein; HOMA-IR, homeostasis model assessment of insulin resistance; hsCRP, high-sensitivity C-reactive protein; LDL, low density lipoprotein; PCOS, polycystic ovary syndrome; SHBG, sex hormone-binding globulin. Data are means ± SD. The effects of group and obesity on continuous variables were analyzed by a two-way general linear model after applying logarithmic transformation when needed. A: *p *< 0.05 for the differences between control women and women with PCOS. B: *p *< 0.05 for the differences between control women and men. C: *p *< 0.05 for the differences between women with PCOS and men.

**Table 2:** Circulating concentrations of metabolic cytokines in the fasting state.

	Control Women		Women with PCOS		Control Men		Group	Obesity	Interaction	
	Non-obese (*n* = 9)	Obese (*n* = 8)	Non-obese (*n* = 9)	Obese (*n* = 8)	Non-obese (*n* = 9)	Obese (*n* = 9)	*P*	*P*	*P*	
Omentin-1 (ng/mL)	215 ± 48	201 ± 75	211 ± 45	196 ± 86	259 ± 47	201 ± 89	0.573	0.044	0.561	
Vaspin (pg/mL)	324 ± 159	249 ± 185	349 ± 482	278 ± 180	169 ± 123	133 ± 61	0.022 ^A,B^	0.472	0.672	
Lipocalin-2 (ng/mL)	108 ± 23	187 ± 72	180 ± 95	150 ± 65	122 ± 54	121 ± 29	0.152	0.203	0.049	
Adipsin (µg/mL)	2.87 ± 0.51	3.46 ± 0.50	3.42 ± 0.89	3.58 ± 0.56	3.20 ± 0.46	3.47 ± 0.84	0.359	0.054	0.533	
PAI-1 (ng/mL)	28.2 ± 15.9	60.0 ± 13.8	33.2 ± 15.0	48.8 ± 22.8	21.2 ± 14.6	44.4 ± 26.6	0.093	< 0.001	0.424	
Chemerin (ng/mL)	8.14 ± 1.54	10.48 ± 2.02	8.49 ± 1.91	9.32 ± 1.30	8.13 ± 1.73	8.53 ± 1.60	0.240	0.015	0.224	
FGF-21 (pg/mL)	392 ± 169	338 ± 144	285 ± 78	349 ± 84	258 ± 128	308 ± 179	0.102	0.489	0.346	
FGF-23 (pg/mL)	312 ± 172	187 ± 41	237 ± 134	195 ± 45	222 ± 111	171 ± 19	0.282	0.013	0.394	

Data are means ± SD. The mean of the three measurements, obtained at fasting before the administration of glucose, lipids and proteins on alternate days, were used for these analyses. The effects of group and obesity on continuous variables were analyzed by a two-way general linear model after applying logarithmic transformation when needed. A: *p *< 0.05 for the differences between control women and men. B:* p *< 0.05 for the differences between women with PCOS and men.

**Table 3:** Correlations between fasting levels and the corresponding AUC after each macronutrient load in all subjects, and according to the presence or absence of obesity.

*Macronutrient*	Omentin-1		Vaspin		Lipocalin-2		Adipsin		PAI-1		Chemerin		FGF-21		FGF-23	
	*r*	*p*	*r*	*p*	*r*	*p*	*r*	*p*	*r*	*p*	*r*	*p*	*r*	*p*	*r*	*p*
**All subjects as a whole**																
*Glucose*	−0.359	0.009	−0.573	<0.001	−0.319	0.021	−0.612	<0.001	−0.567	<0.001	−0.379	0.006	−0.272	0.051	−0.395	0.004
*Lipids*	−0.456	0.001	-0.505	<0.001	−0.249	0.076	−0.661	<0.001	−0.426	0.002	−0.430	0.001	-	-	−0.567	<0.001
*Proteins*	-	-	-0.594	<0.001	−0.460	0.001	−0.512	<0.001	−0.594	<0.001	−0.284	0.041	−0.437	0.001	−0.349	0.011
**Non-obese subjects**																
*Glucose*	-	-	−0.615	0.001	-	-	−0.751	<0.001	−0.490	0.009	-	-	-	-	−0.346	0.077
*Lipids*	−0.589	0.001	−0.688	<0.001	-	-	−0.636	<0.001	-	-	−0.514	0.006	-	-	−0.638	<0.001
*Proteins*	−0.473	0.013	−0.734	<0.001	−0.330	0.092	−0.419	0.030	-	-	-	-	−0.400	0.039	−0.563	0.002
**Obese subjects**																
*Glucose*	−0.456	0.022	−0.590	0.002	−0.455	0.022	−0.418	0.037	−0.657	<0.001	−0.528	0.007	−0.536	0.006	−0.702	<0.001
*Lipids*	-	-	-	-	−0.604	0.001	−0.651	<0.001	−0.502	0.011	−0.370	0.069	-	-	−0.398	0.049
*Proteins*	0.494	0.012	−0.579	0.002	−0.598	0.002	−0.633	0.001	−0.733	<0.001	−0.369	0.069	−0.476	0.016	-	-

*Abbreviations:* AUC, area under the curve. Data were submitted to Pearson’s correlation analysis. Only correlations with *p* < 0.1 are depicted.

**Table 4:** Summary of effects of obesity and group of subjects on fasting and postprandial levels of metabolic cytokines.

Serum Concentrations	Fasting	Postprandial (AUC)
Omentin-1	↓ in obese	↓ after all macronutrient loads (mainly in non-obese after proteins)
Vaspin	↓ in men	↓ during lipid challenge and ↓↓ during protein load Global effect of obesity (smaller response in obese)
Lipocalin-2	↑ in obese only in control women PCOS > control women only in non-obese	↓ during glucose and protein loads
Adipsin	Trends towards ↑ in obese	↓ after all macronutrient loads
PAI-1	↑↑ in obese	↓ during lipid challenge and ↓↓ during protein load Global effect of obesity (larger response in obese)
Chemerin	↑ in obese	↓ during glucose and lipid challenges
FGF-21	-	↑ during glucose, ↓ during lipid challenge and ↓↓ during protein loads
FGF-23	↓ in obese	↓ during protein load, only in non-obese individuals

Abbreviations: ↑ increase; ↓ decrease; > higher; AUC, area under the curve; PCOS, polycystic ovary syndrome.

## Supplementary Materials

The following are available online at https://www.mdpi.com/2072-6643/11/11/2566/s1, Supplementary Data Text.
