# Supplementary material for: Metabolic Cytokines at Fasting and During Macronutrient Challenges: Influence of Obesity, Female Androgen Excess and Sex
Source: Nutrients. 2019 Oct 24;11(11):2566. doi: 10.3390/nu11112566 (PMC6893420; doi:10.3390/nu11112566)
Supplement: Supplementary file 1 [file nutrients-11-02566-s001.pdf]

Supplementary data text

Oral macronutrient challenges

On alternate days, we submitted patients to oral loads of glucose, lipids and protein. The quantities and volumes in the oral loads were adjusted for a total caloric intake of 300 kcal each, irrespective of the macronutrient being administered. Therefore, we administered 200 ml of a 37.5 g/dl glucose solution (GlycoSull Naranja 75 g, Química Clínica Aplicada, Spain) for the oral glucose load, 66 ml of a 4.5 kcal/ml long-chain triglyceride enteral nutrition supplement (Supracal neutro, Nutricia S.R.L., Spain) for the oral lipid load, and 75 g of an enteral nutrition supplement containing caseinates (Proteína NM, Nutrición Médica S.L., Spain) for the oral protein load. The composition of the lipid supplement was 10.6%, 60.8% and 28.6% of saturated, mono, and poly unsaturated fatty acids, respectively. Whereas we observed no problem with the ingestion of the glucose and lipid supplements, the high viscosity and relatively poor palatability of the protein supplement made difficult the administration and ingestion of the same amounts of kcal in all the subjects. Hence, we calculated the amounts of kcal ingested in 21 consecutive protein preparations, after discounting from the initial amount the quantities lost in the preparation and the remnant that was not ingested, resulting into a mean of 304.7 kcal ingested per subject with a 0.05 coefficient of variation.

Multi-analyte profiling of metabolic cytokines

Six adipokines and two fibroblast growth factor family members were determined in serum samples at fasting and during the postprandial phase (60 and 120 min after ingestion of glucose and proteins, or 120 and 240 min after the ingestion of lipids) on the Luminex Magpix system (Luminex Technologies, Austin, USA). The multiplex kits used were Human Adipokine Magnetic Bead Panel 1 (HADK1MAG-61K, including Adipsin, Lipocalin-2 and total PAI-1) (EDM Millipore Corporation, Massachusetts, USA), Human Metabolic Panel 2 (171-AMR2CK, including Omentin-1, Vaspin, FGF-21 and intact FGF-23) (BIORAD, California, USA), and Human Premixed Multi-Analyte Kit (LXSAHM-5, including Chemerin) (R&D, Minneapolis, USA). The lower limits of detection and intra- and inter-assay coefficients of variation were, respectively, 4.8 pg/ml, 2% and 6% for adipsin; 1.7 pg/ml, 4% and 12% for lipocalin-2; 4.1 pg/ml, 5% and 14% for PAI-1; 6.4 ng/ml, 13% and 15% for omentin-1; 1.4 pg/ml, 10% and 13% for vaspin; 69 pg/ml, 10% and 15% for chemerin; 11 pg/ml, 16% and 19% for FGF-21, and 5.8 pg/ml, 10% and 16% for FGF-23. The assays were performed according to the manufacturer's instructions and analyzed using Milliplex Analyst software version 5.1 (EDM Millipore Corporation, Massachusetts, USA).
